# Synergy of Phospholipid—Drug Formulations Significantly Deactivates Profibrogenic Human Hepatic Stellate Cells

**DOI:** 10.3390/pharmaceutics11120676

**Published:** 2019-12-12

**Authors:** Gina Valentino, Cristina Zivko, Florian Weber, Lorine Brülisauer, Paola Luciani

**Affiliations:** 1 Institute of Pharmacy, Friedrich Schiller University Jena, Otto Schott Strasse 41, 07745 Jena, Germany; 2 Department of Chemistry and Biochemistry, University of Bern, Freiestrasse 3, 3012 Bern, Switzerland

**Keywords:** liver fibrosis, essential phospholipids, hepatic stellate cells, liposomes, silymarin, antifibrotic, reversion, quiescent, inactivation

## Abstract

The pivotal role of hepatic stellate cells (HSCs) in orchestrating the bidirectional process of progression and regression of liver fibrosis makes them an ideal target for exploring new antifibrotic therapies. Essential phospholipids (EPLs), with their polyenylphosphatidylcholine (PPC) fraction, either alone or combined with other hepatoprotective substances such as silymarin, are recommended in hepatic impairment, but a scientific rationale for their use is still lacking. Herein, we compared the ability of EPLs to restore quiescent-like features in HSCs with that of dilinoleoylphosphatidylcholine (DLPC), PPC fraction’s main component. Specifically, we screened at the cellular level the antifibrotic effects of PPC formulations in the presence and absence of silymarin, by using LX-2 cells (pro-fibrogenic HSCs) and by assessing the main biochemical hallmarks of the activated and deactivated states of this cell line. We also proved the formulations’ direct effect on the motional order of cell membranes of adherent cells. LX-2 cells, examined for lipid droplets as a quiescence marker, showed that PPCs led to a more prominent deactivation than DLPC. This result was confirmed by a reduction of collagen and α-SMA expression, and by a profound alteration in the cell membrane fluidity. PPC–silymarin formulations deactivated HSCs with a significant synergistic effect. The remarkable bioactivity of PPCs in deactivating fibrogenic HSCs paves the way for the rational design of new therapeutics aimed at managing hepatic fibrosis.

## 1. Introduction

Liver fibrosis is the formation of a pathological amount of scar tissue in the liver due to an increased deposition of extracellular matrix (ECM) proteins in response to chronic injuries [1]. Although the release of ECM proteins is an essential part of the wound-healing response, repeated injuries, as occur in most type of chronic liver diseases (e.g., chronic hepatitis B and C, metabolic disorders, or non-alcoholic fatty liver disease (NAFLD)), promote fibrosis [2]. Advancement of the disease distorts the normal liver parenchyma, resulting in cirrhosis and ultimately compromising liver functionality irreversibly [2].

Hepatic fibrosis usually commences unobserved, and most of the related mortality and morbidity occur after the development of cirrhosis [3]. Chronic liver diseases, with more than 800 million people affected worldwide, represent a major concern for public health [4]. Globally, the mortality rate is approximately 2 million per year due to related complications [3]. Indeed, cirrhosis and liver cancer account for 3.5% of all deaths worldwide, making them the 11th and 16th most common causes of death each year, respectively [5]. The hepatic fibrosis stage is the most important determinant of patient mortality [6], yet to date, there is an unmet medical need for effective anti-fibrotic treatments [7,8,9].

Tissue fibrogenesis was long thought to be a unidirectional, progressive process, but it was found to be potentially reversible when the provoking event is treated or resolved [10]. Although the underlying trigger for fibrosis development is multicausal [2], hepatic stellate cells (HSCs) have been identified as the central disease mediators, as well as the major fibrogenic cell type in liver fibrosis [11]. As a result of chronic hepatic injury, HSCs undergo a complex phenotypic transdifferentiation known as “activation”, responsible for a cell transition from a quiescent status to a fibrogenic, highly proliferative, myofibroblast-like status [11,12]. Quiescent HSCs are characterized by an abundance of cytoplasmic lipid droplets used to store the majority of retinoid present in the body [13]. Activated HSCs experience a loss of lipid droplets, resulting in reduced retinoid storage, an increased ECM protein production (e.g., collagen type I), and an enhanced contractility accompanied by an elevated expression of cytoskeleton filaments (e.g., α-smooth muscle actin (α-SMA)) [13,14,15]. Interestingly, previous studies have established that activated HSCs can undergo apoptosis or reversion to a quiescence-like phenotype [16]. However, the upstream endogenous processes that direct myofibroblasts towards reversion or death are not clear. Progress in gaining insight into this pathogenesis has been made also through the use of immortalized human HSCs (LX-2 cells). LX-2 cells greatly retain key features of activated human HSCs, including retinoid metabolism and fibrogenesis [17]. This cell line can be quiesced or perpetuated in activation [1,11,15,18], providing an important tool to study the mechanisms of action of antifibrotic compounds [19]. The HSCs’ pivotal role in liver fibrosis, their ability to reverse to quiescence, and their easily accessible location in the space of Disse, between blood vessels and hepatocytes, make these cells an important therapeutic target for the disease [2,20].

At present, no effective anti-fibrogenic therapy is available. Since the late 90s, administration of essential phospholipids (EPLs), highly purified extracts of soybeans, has been an approach for the management of fatty liver disease, due to their supposedly antioxidant and anti-inflammatory action [21]. However, the exact mechanism through which EPLs exert their beneficial hepatoprotectant effect is still unknown [21,22]. EPLs are >72% composed of a mixture of polyenylphosphatidylcholines (PPCs), in which 1,2-dilinoleoylphosphatidylcholine (DLPC) is the quantitatively dominant lipid [21]. Phospholipids are generally recognized as safe (GRAS) excipients of the Federal Food, Drug, and Cosmetic Act, and are characterized by high bioavailability [23]. In Eastern European countries, EPLs are recommended for people with non-alcoholic steatohepatitis (NASH) [22], and in 2017, EPLs alone and combined with other active ingredients (e.g., herbal preparation, vitamins) accounted for 46.9% of the hepatoprotective drug market in Russia [22]. Products such as Livolin^®^, Essenvita^®^, or Essentiale^®^ are indicated as nutritional support in the management of liver fatty degeneration, hepatitis (caused by toxins, medicines, or alcohol abuse), or cirrhosis. Among all hepatoprotective substances incorporated into EPL formulations, silymarin is one of the most frequently investigated [24]. The therapeutic action of the lipophilic extract from the seeds of *Silybum marianum*, collectively known as silymarin, in chronic liver disease seems to be related to a reduction of HSC activation in primary culture, along with inhibition of proinflammatory cytokine production and antioxidant effects [25]. Recent evidence highlights silymarin’s inhibitory effect on LX-2 cells fibrogenic gene expression [26]. SILIPHOS^®^, Hepatrine^®^, and Karsil^®^ are three examples of commercially available silymarin-based products. However, to our knowledge, a systematic investigation on the antifibrogenic effect of a combination between PPC and silymarin screened at a cellular level has not been carried out to date.

Controversially, the exact mechanism of the hepatoprotective action of PPCs, attributed to DLPC, the pivotal component of PPCs [21,27], remains unclear in humans, and more rigorous clinical trials are required to determine the benefits in liver fibrosis. Studies have documented PPCs to be protective against liver damage caused by chronic alcohol consumption in patients with hepatitis B [21] or primates with fibrosis [28], and against non-alcoholic hepatic fibrosis induced by fat diet, albumin, or carbon tetrachloride (CCl_4_) in rats [29,30]. One animal study in rats also demonstrated that EPLs alleviate the accumulation of fat in the steatosis induced by orotic acid [31]. PPC-based treatments resulted in a reduced accumulation of collagen in the culture media of rat acetaldehyde-activated HSCs as a model for alcoholic disease [32]. The involved mechanisms could be in part attributed to DLPC stimulation of collagenase activity, responsible for the breakdown of collagen [33,34]. Other studies have also reported PPC-mediated suppression of collagen and α-SMA expression in human HSCs by showing reduced levels of reactive oxygen species induced by TGF-β_1_ [35]. PPCs have been reported to possess an intriguing ability to incorporate into damaged sections of hepatic cell membranes, helping to maintain membrane fluidity and function [36]. PPC supplementation is correlated, among other effects, with amelioration of ethanol-induced decreases in membrane-bound enzyme activities (e.g., phosphatidylethanolamine methyltransferase) and with an adjustment of phospholipid depletion [37]. This could be one of the mechanisms through which PPCs exert their antifibrotic effect. However, studies on PPCs in hepatic fibrosis to date do not permit an optimization of EPL’s therapeutic utility in liver fibrosis because of the lack of long-term controlled clinical trials aimed at determining their precise benefit (e.g., for inducing histological changes, alleviating symptoms, and slowing the progression of the disease). A comprehensive, standardized knowledge of their role in liver fibrosis and their mechanism of action on human HCSs is still missing.

The aim of this study was to provide a quantitative assessment at the cellular level of the antifibrotic potential of various phospholipid-based drug formulations in an in vitro model of hepatic fibrosis. Specifically, we chose a dynamic fibrogenic cell model of human HSCs that can be either quiesced, or further activated and then reverted to the quiescent-like status. By means of this HSC transdifferentiation model, the antifibrotic properties of PPC-based formulations were evaluated.

To gain a better understanding of PPCs’ mechanism of action, a PPC-rich (>75%) lipid, S80, and its corresponding complexes with MgCl_2_ and CaCl_2_ salts (SMg and SCa, respectively) were formulated as liposomes, tested on HSCs, and compared. Furthermore, formulations of either DLPC, DOPC, or DOPC/DLPC, mirroring the DLPC content in the employed PPC-rich lipids, were investigated. Additionally, the coadjuvant effect of the hepatoprotectant silymarin co-formulated with PPC was studied. In order to define the molecular mechanism underpinning the antifibrotic effect of PCCs, we tested the expression level of lipid droplets and that of main fibrotic markers (i.e., α-SMA, collagen). We finally made innovative use of fluorescence anisotropy to study the motional order of cell membranes of living LX-2 cells.

## 2. Materials and Methods

### 2.1. Materials

Soybean phospholipid with 75% phosphatidylcholine (S80), soybean phospholipid 80% complexed with MgCl_2_ (S 80 M; SMg), soybean phospholipid 80% complexed with CaCl_2_ (S 90; SCa), and 1,2-dioleoyl-sn-glycero-3-phosphocholine (DOPC) were a kind gift from Lipoid GmbH (Ludwigshafen, Germany). Their fatty acid content is available in the Supporting Materials (Table S1). 1,2-dilinoleoyl-sn-glycero-3-phosphocholine (DLPC) was purchased from Avanti Polar Lipids (Alabaster, AL, USA). Membranes for liposome extrusion were from Whatman^®^ Nucleopore^TM^ (Maidstone, UK). The LX-2 cells immortalized human hepatic stellate cell line, Dulbecco’s Modified Eagle Medium (DMEM) (4.5 g/L glucose, with phenol red, no glutamine, no sodium pyruvate), penicillin/streptomycin mixture (penicillin: 10,000 U/mL, streptomycin: 10,000 μg/mL), l-glutamine (200 mM), fetal bovine serum (FBS), Accutase^®^, and TGF-β_1_ were purchased from Merck Millipore (Darmstadt, Germany). Silymarin, Oil red O (ORO; 0.5% *w*/*v* in propylene glycol), retinol (Rol), palmitic acid (PA), Cell Counting Kit-8 (CCK-8), 4’,6-diamidino-2-phenylindole (DAPI), diphenylhexatriene (DPH), (1-(4-trimethylammoniumphenyl)-6-phenyl-1,3,5-hexatriene *p*-toluenesulfonate) (TMA-DPH), bovine serum albumin (BSA), and dimethyl sulfoxide (DMSO) were bought from Sigma-Aldrich (Schnelldorf, Germany). Sirius Red/Fast Green collagen staining was obtained from Chondrex (Redmond, WA, USA), α-smooth muscle actin (α-SMA) monoclonal antibody, and Alexa Fluor^®^ 647-labeled goat anti-mouse IgG from Thermo Fisher Scientific (Waltham, MA, USA), and DMEM (4 mM L-glutamine, 4.5 g/L glucose, with sodium pyruvate, no phenol red) from HyClone (Logan, UT, USA). Sodium hydrogen phosphate (Na_2_HPO_4_), potassium dihydrogen phosphate (KH_2_PO_4_), sodium chloride (NaCl), potassium chloride (KCl), Roti^®^-Histofix 4% (acid free, pH 7.4% *w*/*v* phosphate-buffered formaldehyde solution), formaldehyde (37% *w*/*v* in H_2_O), chloroform (CHCl_3_), ethanol (EtOH), trifluoroacetic acid (TFA), methanol (MeOH), acetonitrile (ACN), acetic acid, and 4-(2-Hydroxyethyl) piperazine-1-ethanesulfonic acid sodium (HEPES) salts were purchased from Carl Roth (Karlsruhe, Germany). Cell culture plates were from Greiner Bio One International GmbH (Monroe, NC, USA).

### 2.2. Development of PPC-Based Formulations

Lipid vesicles containing either synthetic phosphatidylcholines (DOPC, DLPC, or a mixture DOPC/DLPC (57:43 mol%)) or a natural soybean-derived phospholipid (S80, SMg, or SCa) were prepared according to the film hydration extrusion method [38]. Briefly, an appropriate aliquot of lipid stock solution in CHCl_3_ was evaporated under a stream of nitrogen until dry. Traces of solvent were further removed by keeping the resulting thin lipid films under vacuum overnight. After hydration with HEPES buffer (10 mM in H_2_O, pH 7.4), the liposomal formulations (final lipid concentration 50 mM) were extruded 10 times through a 0.2 µm polycarbonate membrane at room temperature (RT) using a LIPEX^®^ extruder (Transferra Nanosciences Inc., Burnaby, B.C., Canada). Lipids were quantified chromatographically as detailed in the Supporting Materials and Methods.

### 2.3. Development of Silymarin PPC-Based Formulations

Silymarin liposomes were produced with S80, SMg, SCa, or DOPC starting from a dry lipid film (vide supra). Aliquots of methanolic stock solution of silymarin were added to the lipid film to obtain a final theoretical lipid-to-silymarin ratio of 1:11. After removal of the organic solvent, the resultant lipid film with silymarin was hydrated with HEPES buffer (10 mM, pH 7.4), and the liposomal formulation was extruded as described in Section 2.2. Lipids and silymarin were quantified as detailed in the Supporting Materials and Methods.

### 2.4. Characterization of Particle Size and Size Distribution

The hydrodynamic diameter and the size distribution (polydispersity index, PDI) of the liposomes were measured with the Zetasizer Nano ZS (Malvern, Worcestershire, UK) with a 173° backscatter angle and a 633 nm helium–neon laser. The produced liposomes were diluted with MilliQ water to a concentration of 0.2 mM. The intensity size distribution of the liposomes was typically unimodal; therefore, the autocorrelation function was analyzed according to the cumulant method.

### 2.5. Cell Culture and General Design of Cell Experiments

LX-2 cells were grown at 37 °C in a humidified atmosphere containing 5% CO_2_ in complete medium (CM): DMEM (4.5 g/L glucose, phenol red, no L-glutamine, no sodium pyruvate) supplemented with 1% *v*/*v* penicillin/streptomycin mixture (penicillin: 10,000 U/mL, streptomycin: 10,000 μg/mL), 1% *v*/*v* of L-glutamine (200 nM), and 2% *v*/*v* FBS. Subcultivation was performed with Accutase at a cell confluency of about 80% according to the manufacturer instructions. Cells at passage number 5 to 18 were used for cell experiments.

Cell experiment medium (EM) was serum-free, prepared with DMEM and supplemented with 1% *v/v* penicillin/streptomycin and 1% *v*/*v* L-glutamine. For the cell proliferation assay, serum-free and phenol-red-free DMEM supplemented with 1% *v/v* penicillin/streptomycin was used instead. 

For experiments, LX-2 cells were seeded either in 12 well microtiter plates with 1 mL CM/well at a density of 100,000 cells/well, or in transparent or black 96 well microtiter plates with 100 μL CM/well at a density of 12,000 cells/well, and cultured 18 h at 37 °C, 5% CO_2_ to 70–90% confluency. Treatments were always performed with 1 mL/well for 12 well plates or 100 μL/well for 96 well plates at 37 °C, 5% CO_2_.

### 2.6. Experimental Approach: Direct Treament of LX-2 Cells and Treatment of Perpetuated LX-2 Cells (pLX-2 Cells)

**Scheme 1:**
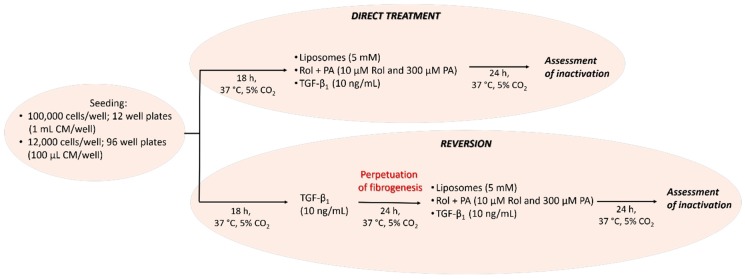
Study design of the progression and regression of fibrogenesis in vitro.

After cell seeding, the CM from seeded LX-2 cells was discarded and the cells were rinsed once with phosphate-buffered saline (PBS). Treatments of LX-2 cells in microtiter plates were then performed directly on naïve LX-2 cells or after a further activation of LX-2 cells (perpetuated LX-2 cells; pLX-2 cells) obtained with TGF-β_1_ [12,39,40]_._ For the direct treatment, the formulations were mixed with EM and LX-2 cells were incubated with this solution for 24 h, as described below. For the perpetuation, TGF-β_1_ in PBS (with 0.1% *w*/*v* BSA; 10 µg/mL) was diluted in EM to reach a final TGF-β_1_ concentration of 10 ng/mL. LX-2 cells were then treated with this solution for 24 h [41,42]. TGF-β_1_ was discarded, and the cells were rinsed once with PBS and then treated as described below. TGF-β_1_ (10 ng/mL) was also used as control to induce fibrogenesis in the direct treatment starting from naïve LX-2 cells [12,39,40]_._ A graphical representation of the experimental approach is depicted in Scheme 1.

### 2.7. Induction of Quiescent-Like LX-2 Cells with Retinol and Palmitic Acid (Rol + PA)

LX-2 cells or pLX-2 cells were incubated for 24 h with retinol and palmitic acid (Rol + PA) to reverse LX-2 cell fibrogenesis (Scheme 1) following previously reported results [43]. Rol + PA stock solutions were vigorously mixed with EM to reach 10 μM Rol and 300 μM PA. Wells with vehicle control received equal volumes of EM.

### 2.8. LX-2 Cells and pLX-2 Cells Treatment with PPC-Based Formulations

Each liposomal formulation was mixed with EM to obtain a medium with 5 mM lipid concentration (LM). LX-2 cells or pLX-2 cells were then incubated with LM for 24 h (Scheme 1) to cover the same incubation range as Rol + PA and TGF-β_1_ treatment.

Wells with vehicle control received equal volumes of EM. To assess the antifibrotic effect of the treatment on the cells, the presence of cytosolic lipid droplets and collagen, as well as the expression of α-SMA, were analyzed as described below.

### 2.9. Analysis of Lipid Droplet Content

After cell treatment, LX-2 cells in 12 well plates were washed three times with PBS, fixed with 500 μL/well Roti^®^-Histofix 4% for 15 min at RT, and washed once with 1 mL/well deionized MilliQ water. To avoid artefactual formation of lipid droplets due to ORO solvents such as ethanol and isopropanol [44], cells were stained with a 0.5% *w*/*v* ORO solution in propylene glycol (500 μL/well) for 15 min at RT. The staining was carefully washed off using PBS. Nuclei were then counterstained with a 3.6 µM DAPI solution in PBS for 5 min at RT (300 mL/well). Afterwards, cells were rinsed with PBS (1 mL/well). Fluorescence and phase contrast image acquisition was performed using a Nikon Ti-U (Nikon Instruments, Melville, NY, USA) inverted microscope (acquisition details in the Supporting Materials and Methods). The fluorescent binary area and the object count were detected with the NIS Elements (Nikon Instruments, Melville, NY, USA) software v. 5.00 and exported.

### 2.10. Analysis of Fibrillar Collagen

After cell treatment in 12 well plates, the total protein content and the collagen content were measured with the Sirius Red/Fast Green collagen staining kit according to the manufacturer’s instructions. Briefly, LX-2 cells were carefully washed three times with PBS and then fixed for 15 min at RT with a freshly prepared Kahle fixative solution (60 mL distilled H_2_O, 28 mL 96% *v/v* EtOH, 10 mL 37% *w*/*v* formaldehyde, 2 mL glacial acetic acid) (500 μL/well). After rinsing cells with PBS, Sirius Red/Fast Green staining solution was added (200 μL/well) and cells were left at RT for 30 min. The cells were then washed three times with PBS (500 μL/well), fresh PBS was added (1 mL/well), and bright field images were acquired using a Nikon Ti-U inverted microscope (acquisition details in the Supporting Materials and Methods).

### 2.11. Analysis of α-SMA Expression

After cell treatment in 12 well plates, cells were washed twice with PBS and then fixed and permeabilised in 500 μL/well ice-cold MeOH for 30 min at RT. After fixation, cells were washed once with PBS and incubated with 500 μL/well blocking solution (5% *w*/*v* BSA in PBS) for 30 min at RT. Primary mouse IgG diluted in blocking solution (0.1 μg IgG/mL) was incubated with cells (500 μL/well) for 18 h at RT. After three washes with blocking solution, cells were incubated with secondary goat anti-mouse IgG-Alexa Fluor 647 diluted in blocking solution (10 μg/mL) for 2 h at RT (500 μL/well). Plates were washed three times with PBS, stained with 3.6 µM DAPI solution for 5 min, and washed again three times with PBS. Fluorescence images acquisition was performed using a Nikon Ti-U inverted microscope (acquisition details in the Supporting Materials and Methods). The fluorescent binary area and the object count were detected with the NIS Elements software v. 5.00 and exported.

### 2.12. Cell Proliferation Assay

The CCK-8 assay was used following the manufacturer’s instruction. Briefly, after treatments (100 μL/well; 96 well plate), cells were washed twice with PBS. A volume of 90 μL of serum-free and phenol-red-free DMEM supplemented with 1% *v/v* penicillin/streptomycin and a volume of 10 μL of CCK-8 were added to each well. LX-2 cells were incubated for further 2 h at 37 °C, 5% CO_2_. Afterwards, the absorbance was measured at 450 nm using a plate reader (Spark 10M, Tecan, Switzerland) at 37 °C. The following equation was used to calculate the cell metabolic activity in percent (Equation (1)):

Cell metabolic activity (%) = (OD sample/OD control) × 100,(1)

where “OD sample” refers to the optical density of the cells treated with the substances and “OD control” is the cells exposed to phenol-red-free DMEM supplemented with 1% *v*/*v* penicillin/streptomycin only.

### 2.13. Motional Order of the Cell Membrane in Adherent LX-2 Cells

DPH and TMA-DPH were dissolved in DMSO to a concentration of 8 and 5 mM, respectively, and were stored at −20 °C, protected from light. DPH and TMA-DPH stock solutions in DMSO and working solutions of DPH (8 μM) or TMA-DPH (5 μM) in PBS were prepared fresh for each experiment. After cell treatment (100 μL/well), the cells were washed three times with PBS and 100 μL of DPH-PBS (8 μM) or TMA-DPH-PBS (5 μM) were added to each well of the 96 well plate (black bottom and wall). LX-2 cells were then further incubated for either 2 h at 37 °C, 5% CO_2_ with DPH–PBS, or for 10 min at 37 °C, 5% CO_2_ with TMA-DPH–PBS. After one PBS wash (100 μL/well), all of the remaining solution was aspirated from the wells. The fluorescent anisotropy was measured with a Tecan’s Spark 10M plate reader equipped with polarization filters (monochromator mode, λ_ex_ = 360 nm, λ_em_ = 430 nm) and calculated by applying the following formula (Equation (2)):

(2) $$r=\frac{G\times I_{∥}-I_{⊥}}{G\times I_{∥}+2I_{⊥}},$$

where *G* was 1.678 for DPH and 1.559 for TMA-DPH.

### 2.14. Statistical Analysis

All experiments were performed in at least three independent replicates, and samples were freshly prepared if not stated otherwise.

One-way ANOVA analysis of variance was used to compare means of independent experiments. Significant differences in lipid droplets quantification, α-SMA expression, and motional order of cell membrane among treatments were compared by Tukey’s multiple comparisons test (**** *p* ≤ 0.0001, *** *p* ≤ 0.001, ** *p* ≤ 0.01, *p ≤ 0.05). Data are presented as mean  ±  S.D (standard deviation calculated from independent samples).

## 3. Results and Discussion

### 3.1. Progression and Regression of Fibrogenic Features in LX-2 Cells

As previously described (Scheme 1), in our study cells were treated with TGF-β1 and Rol + PA in order to thoroughly investigate the progression and regression of LX-2 cells’ fibrogenic feature. The results were in agreement with the expected behavior of activated and quiescent-like HSCs, respectively [39,40,43,45].

Upon treatment with Rol + PA, LX-2 cell proliferation was inhibited in accordance with previous results [45,46], while for LX-2 cell perpetuation, the exposure to 10 ng/mL TGF-β1, a major fibrogenic cytokine in liver disease, resulted in a typical increase in cell proliferation (Figure S1) [47].

After assaying the metabolic activity, the in vitro model was characterized by monitoring lipid droplet storage along with collagen and α-SMA expression as key markers. Although the use of free fatty acids (i.e., palmitic acid, or a mixture palmitic/oleic acid) can result in the activation of HSCs grown in monoculture or in simultaneous co-culture with hepatocytes [48,49], it has been reported that palmitic acid or oleic acid supplemented with retinol significantly induce changes in lipid droplets and promote the reversion of activated HSCs [50]. In a previous study, the enlarged droplets obtained with retinol and oleic acid treatments resulted in the recruitment of ATG2A protein to downregulate autophagy and reverse the activation of LX-2 cells [50]. The presence of lipid droplets in HSCs is notably influenced by dietary retinol intake [51,52]. Generally, retinol binds cellular retinol-binding proteins (CRBPs), and it is converted to retinyl esters by the retinol acyltransferase (LRAT) [53]. In the activated state, retinol is also oxidized to retinoic acid which binds to Rarα proteins, upregulating the expression of CRBPs, and therefore increasing the esterification process [53].

**Figure 1:**
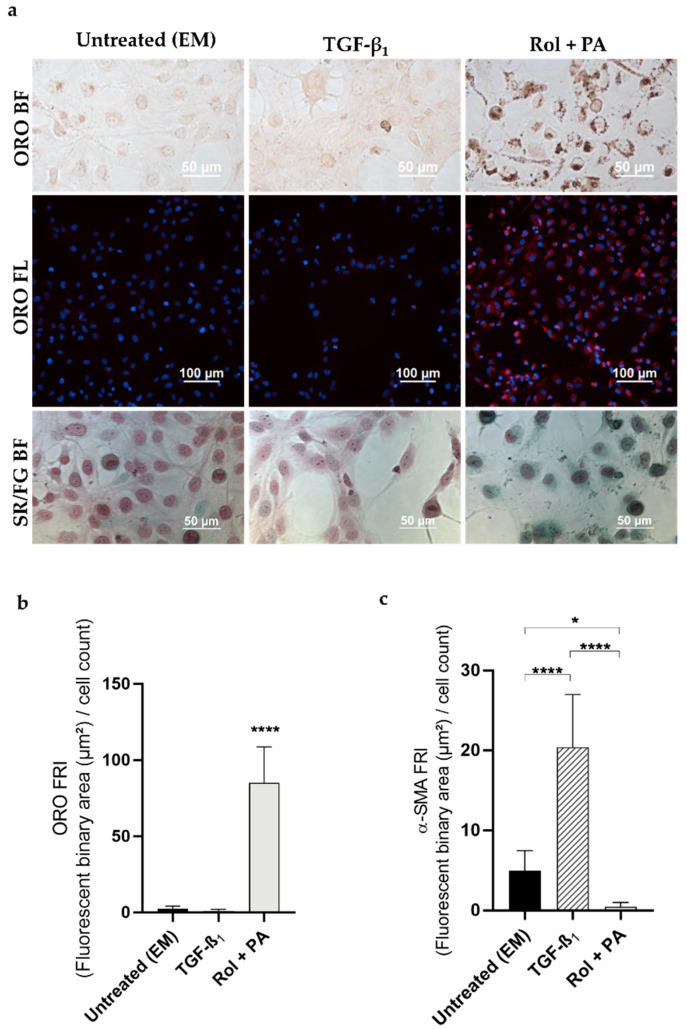
Effect of TGF-β_1_ and Rol + PA on LX-2 cells. (**a**) Representative microscopy images of lipid droplets (ORO staining) and collagen (Sirius Red/Fast Green staining). Lipid droplets in bright field (ORO BF) appear as brown spots and in fluorescence (ORO FL) as red spots (nuclei stained with blue DAPI). The presence of collagen (SR/FG BF) is observed in purple in bright field, while non-collagenous proteins are stained green. (**b**) Bar graph showing the ORO fluorescence normalized to the number of cells in the DAPI field (FRI). **** *p* ≤ 0.0001 vs. EM and TGF-β_1_. Mean ± S.D. (*n* = 3–6). (**c**) Bar graph showing the α-SMA fluorescence normalized to the number of cells in the DAPI field (FRI). * *p* ≤ 0.05, **** *p* ≤ 0.0001. Mean ± S.D. (*n* = 3).

In our study, the combination of Rol and PA stimulated the formation of lipid droplets by an upregulation of the adipose differentiation-related (ADRP) protein, indicating LX-2 cell quiescence, as well evidenced in an earlier research [43]. Within the evaluated time frame, Rol + PA treatment induced a rise in the number of lipid droplets visible as brown spots in bright field and red spots in fluorescence images in comparison to what was observed in untreated LX-2 cells (EM) or TGF-β_1_-treated cells (Figure 1a).

Beside the qualitative interpretation, the quantitative analysis of fluorescence in images maximized the amount of information extracted and enabled a more transparent observation of the different treatment effects on LX-2 cells. A similar analysis was previously reported [54] but, for comparative purposes, our data were treated differently. Briefly, for each image, a fluorescent (ORO or and α-SMA) relative intensity (FRI) value was obtained by normalizing the fluorescent binary area (µm^2^) in the fluorescent field to the number of objects in the DAPI field. Rol + PA treatment induced a 30-fold increase in FRI of lipid droplets in the cytoplasm compared to untreated LX-2 cells (EM).

LX-2 cell staining with Sirius Red/Fast Green showed purple-stained collagen fibers following EM and TGF-β_1_ treatments, clearly indicative of activated cell status [39]. In contrast, quiescent Rol + PA-treated cells displayed a predominance of green non-collagen proteins (Figure 1a).

The quantitative immunocytochemical analysis of the expression of the intermediate α-SMA filament proteins confirmed what was observed with the collagen staining. Exposing HSCs to TGF-β_1_ induced a 4-fold increase in the expression of the α-isotype of actin, which has been reportedly associated with early stage liver fibrogenesis in experimental models and to human fibrosis in chronic liver disease [55] (Figure 1c). The change in FRI from 4.9 (EM sample) to 20.4 (TGF-β_1_ treatment) proved the perpetuation of LX-2 cell activation. The increase is known to lead to biochemical modifications and render the fibrotic liver ever-increasingly less susceptible to remodeling and repair until a point of no return is reached [2].

**Figure 2:**
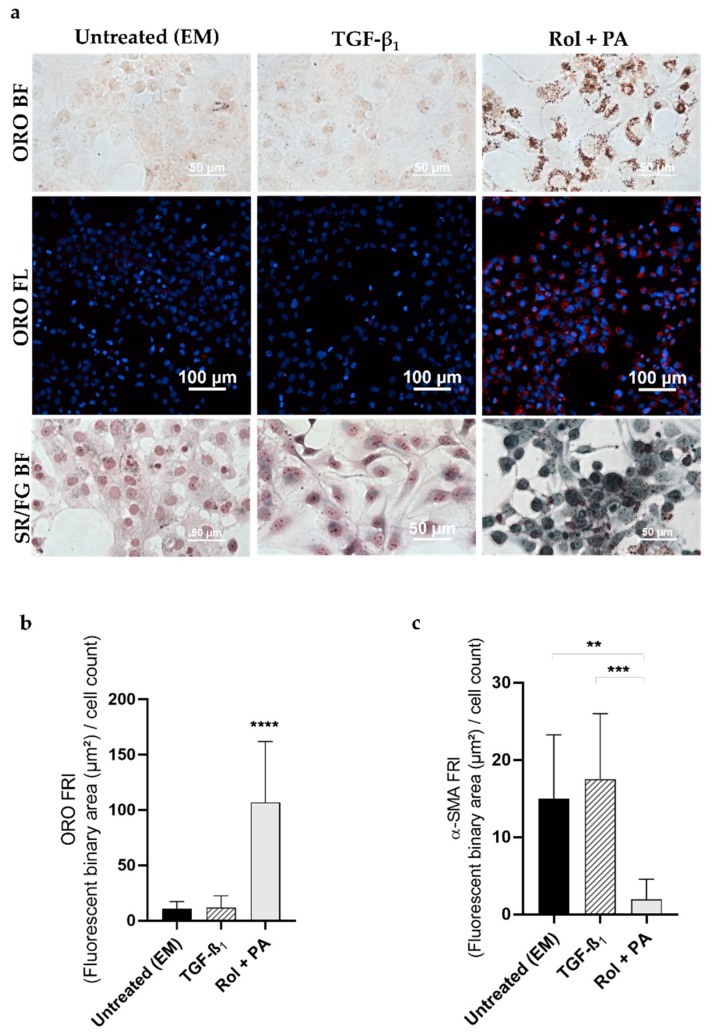
Effect of TGF-β_1_ and Rol + PA on pLX-2 cells. (**a**) Representative microscopy images of lipid droplet staining (ORO in bright field and fluorescence; ORO BF; ORO FL) and collagen staining (Sirius Red/Fast Green in bright field; SR/FG BF). (**b**) Bar graph showing the ORO fluorescence normalized to the number of cells in the DAPI field (FRI). Mean ± S.D. (*n* = 3–6). **** *p* ≤ 0.0001 vs. EM and TGF-β_1_. (**c**) Bar graph showing the α-SMA fluorescence normalized to the number of cells in the DAPI field (FRI). Mean ± S.D. (*n* = 3). ** *p* ≤ 0.01, *** *p* ≤ 0.001.

However, the reversibility of HSCs in a TGF-β_1_-perpetuated activation (pLX-2 cells) was successfully assessed with Rol + PA. Indeed, pLX-2 cells exposed to Rol + PA showed a notable rise in the number of lipid droplets visible in bright field and fluorescence images (Figure 2a) corresponding to a 10-fold increase of the ORO Rol + PA FRI compared to EM (Figure 2b). There was no remarkable presence of lipid droplets in EM or TGF-β_1_ p-LX-2 cells (Figure 2b).

After LX-2 cell perpetuation, TGF-β_1_–treated cells resulted positive for the presence of collagen, as expected (Figure 2a). Sirius Red/Fast Green staining in bright field revealed purple collagen fibers, characteristic of activated HSCs. In contrast, predominance of non-collagenous proteins was observed in green in Rol + PA-treated pLX-2 cells, indicating reversion of the perpetuated cell activation to a quiescent-like phenotype (Figure 2a).

The expression of α-SMA verified the presence of filaments in EM-treated cells with an FRI shift from 17.5 (TGF-β_1_ treatment) to 15 (EM sample) (Figure 2c). Rol + PA treatments asserted the possibility of reversion with an α-SMA FRI of 2. However, compared to the FRI of Rol + PA-treated naïve LX-2 cells, an FRI of 2 is a 4-fold rise in α-SMA expression. This could be explained by a higher susceptibility of reverted HSCs to recurring fibrogenic stimuli, as observed in previous research [56].

These results show that the phenotype of HSC was successfully modulated by TGF-β_1_ and Rol + PA, and that an informative quantification of typical fibrosis features at the cellular level could be carried out.

The above-described in vitro protocols were further used to screen the impact of our own phospholipid-based formulations on activated LX-2 cells, in absence and presence of the hepatoprotectant silymarin.

### 3.2. In Vitro Antifibrotic Effect of Phospholipid-Based Formulations

Lipid vesicles were produced via the film hydration method as previously reported [38], and the subsequent extrusion through polycarbonate membranes resulted in large vesicles of expected size range and PDI (Figure S2). Lipid quantification via HPLC revealed that the lipid content in the extruded liposomes was approximately 100% (Figures S3 and S4). For the formulations containing silymarin, an encapsulation efficiency in the range 76–93% was observed (Figures S5 and S6).

Liposomes with a total lipid concentration of 5 mM were applied for LX-2 cell treatments to maximize the response concerning lipid droplet presence, covering the same 24 h incubation range as used for Rol + PA-and TGF-β_1_-treated LX-2 cells.

Percentages of growth and cell viability of LX-2 cells following PCC-based antifibrotic treatments were first assessed in order to exclude any possible toxicity. As expected, liposomal treatments with and without silymarin did not impair cell viability (Figure S1). The gain in metabolic activity of the lipids compared to Rol + PA could be related to changes in mitochondrial activity due to lipid metabolism and the formation of a high number of lipids droplets [57]. In our study, silymarin liposomes exerted no remarkable effect on LX-2 cells’ metabolic activity compared to EM-treated cells (Figure S1).

**Figure 3:**
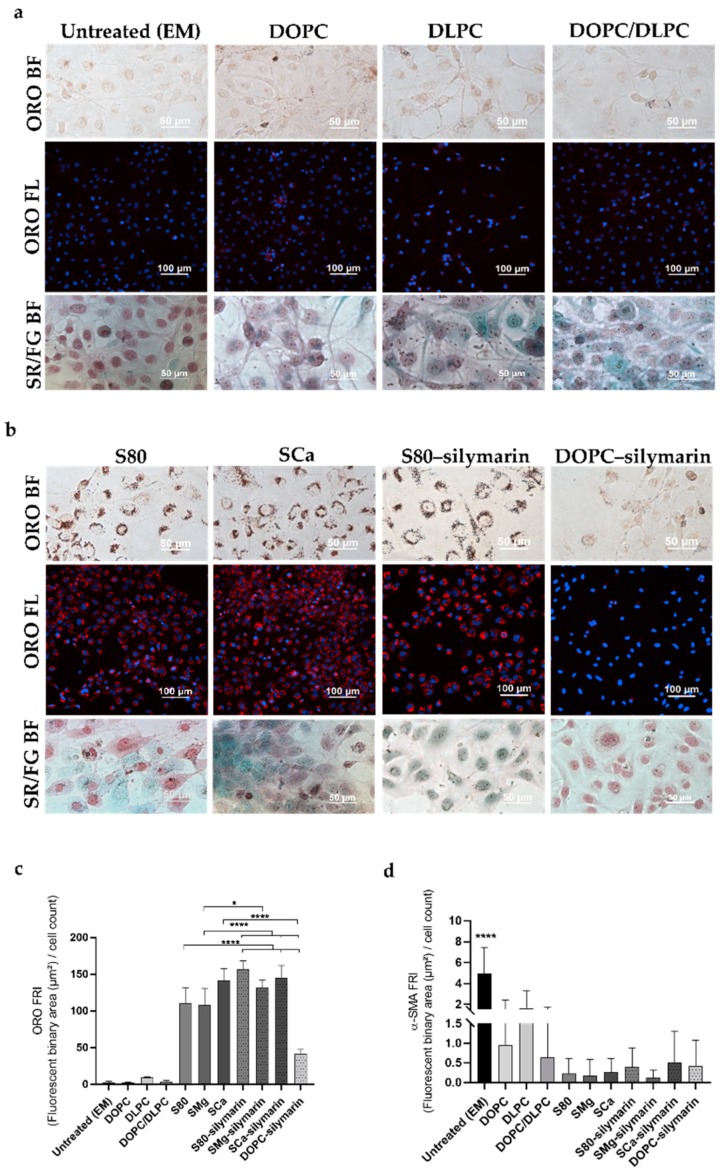
LX-2 cells directly treated with lipid-based formulations. (**a**,**b**) Representative images of ORO in bright field and fluorescence (ORO BF; ORO FL) and Sirius Red/Fast Green staining in bright field (SR/FG BF). (**c**) Bar graph showing the ORO fluorescence normalized to the number of cells in the DAPI field (FRI). Mean ± S.D. (*n* = 3–6). * *p* ≤ 0.05, **** *p* ≤ 0.0001. EM, DOPC, and DLPC/DOPC **** *p* ≤ 0.0001 vs. S80, SMg, SCa, S80–silymarin, SMg–silymarin, SCa–silymarin, and DOPC–silymarin. DLPC **** *p* ≤ 0.0001 vs. S80, SMg, SCa, S80–silymarin, SMg–silymarin, SCa–silymarin, and *** *p* ≤ 0.001 vs. DOPC–silymarin (not shown). (**d**) Bar graph showing the α-SMA fluorescence normalized to the number of cells in the DAPI field (FRI). Mean ± S.D. (*n* = 3). **** *p* ≤ 0.0001 vs. DLPC, DOPC, DOPC/DLPC, S80, SMg, SCa, S80–silymarin, SMg–silymarin, SCa–silymarin, and DOPC–silymarin.

The extent of LX-2 cells’ deactivation to a quiescent-like status was investigated by ORO staining (Figure 3a,b and Figure S7). After 24 h incubation, DLPC-based formulations induced only a negligible increase in the number of HSCs lipid droplets in case of pure lipid or DOPC/DLPC-mixed formulations (Figure 3a). Contrariwise, cytoplasmic lipid droplets could be localized when S80, SMg, and SCa were used in the formulations (110.4 FRI, 108.3 FRI, 141.5 FRI, respectively) (Figure 3b and Figure S7). Those values corresponded to a remarkable 41-fold increase in FRI in comparison with that obtained with EM (2.9 FRI) (Figure 1b) and a 1.4-fold increase even in comparison with the quiescence-inducing Rol + PA standard treatment (87 FRI; Figure 1b). When silymarin was formulated with S80, SMg, and SCa, the ORO staining revealed lipid droplet accumulation in LX-2 cells (Figure 3b and Figure S7) a 49-fold increase in fluorescence originating from the staining fluorescence quantification was detected (156.5 FRI, 131.7 FRI, 145.4 FRI, respectively) (Figure 3c) with respect to untreated cells (EM, 2.9 FRI; Figure 1b). The increase in FRI obtained by formulating silymarin in DOPC (41.9 FRI) resulted in only a 14-fold increase with respect to untreated cells. The co-formulation of a hepatoprotectant with PPCs resulted in an efficient synergy, reverting myofibroblast-like HSCs to their quiescent-like phenotype only when S80 was employed. However, the droplet fluorescence of S80 and SMg showed significant differences toward the FRI from SCa and the formulation with silymarin (Figure 3c). The outstanding antifibrotic properties of the bioactive S80, SCa, and SMg were significantly improved by the addition of silymarin, but not in additive or synergistic fashion.

The effect of PPC-based formulations on collagen fibers, resulting from an imbalance between collagen deposition and reabsorption typical of chronic fibrogenic processes, was then assessed. Treating LX-2 cells with S80, SMg, or SCa, with or without silymarin, led to the formation of well-defined green-stained non-collagen fibers (Figure 3b and Figure S7) similarly to that observed with Rol + PA (Figure 1a). In contrast, when LX-2 cells were treated with DOPC and DOPC–silymarin, purple-stained collagen was prevalently visible (Figure 3a,b). Although DOPC/DLPC and DLPC treatments had no impact on the appearance of lipid droplets, the Sirius Red/Fast Green staining detected the presence of non-collagen fibers mixed with purple collagenous ones following treatments with these lipids (Figure 3a), in accordance with previous results [28].

All liposomal formulations induced a lower expression of α-SMA compared to TGF-β_1_ and EM-treated cells, corresponding approximately to a 20-fold decrease of fluorescence (Figure 3d), confirming the ability of PPC formulations to revert the myofibroblast-like phenotype of naïve LX-2 cells. All treatments resulted in a statistically significant difference (*p* ≤ 0.0001) compared to TGF-β_1_.

Aiming at assessing the reversibility of HSCs in a perpetuated activation, LX-2 cells were pre-treated with TGF-β_1_ for 24 h before exposing them to liposomal formulations.

**Figure 4:**
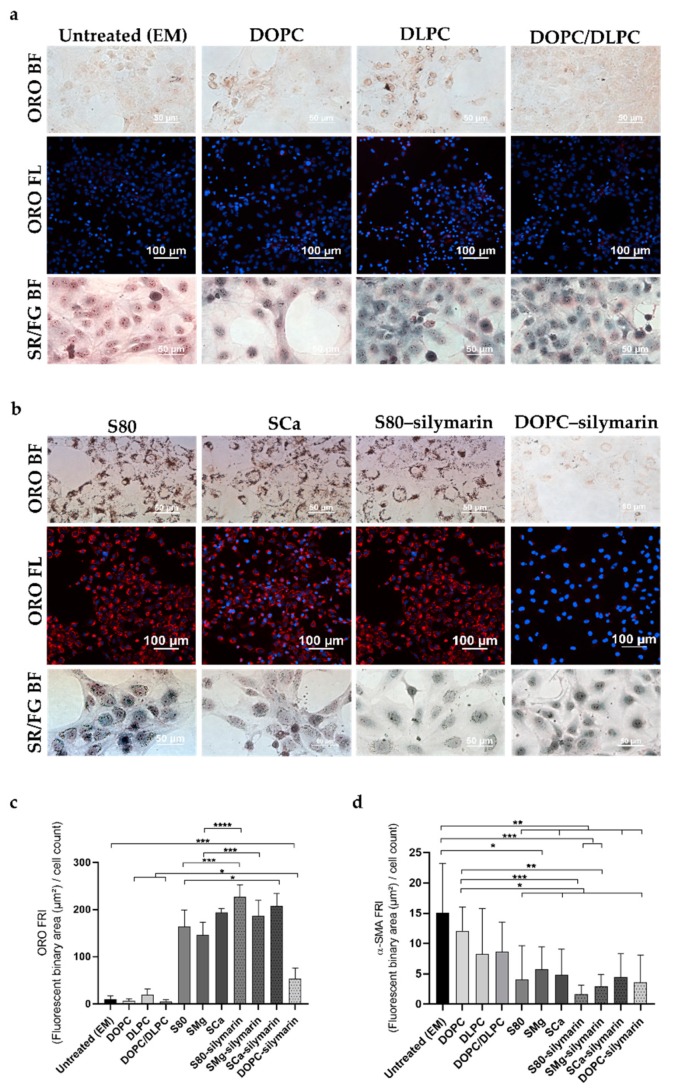
pLX-2 cells treated with lipid-based formulations. (**a**,**b**) Representative images of ORO and Sirius red/Fast green staining in bright field (ORO BF; SR/FG BF) and fluorescence (ORO FL). (**c**) Bar graph showing the ORO fluorescence normalized to the number of cells in the DAPI field (FRI). Mean ± S.D. (*n* = 3–6). * *p* ≤ 0.05, *** *p* ≤ 0.001, **** *p* ≤ 0.0001. Not shown EM, DOPC, and DLPC/DOPC **** *p* ≤ 0.0001 vs. S80, SMg, SCa, S80–silymarin, SMg–silymarin, and SCa–silymarin. EM *** *p* ≤ 0.001 vs. DOPC–silymarin. DOPC and DOPC/DLPC * *p* ≤ 0.05 vs. DOPC–silymarin. (**d**) Bar graph showing the α-SMA fluorescence normalized to the number of cells in the DAPI field (FRI). Mean ± S.D. (*n* = 3). * *p* ≤ 0.05, ** *p* ≤ 0.01, and *** *p* ≤ 0.001.

pLX-2 cells proved to be still sensitive to quiescence-inducing treatments, as shown by the results obtained with the ORO staining (Figure 4a,b and Figure S8). Analogously to what was observed with naïve LX-2 cells, treating pLX-2 cells with S80, SMg, and SCa liposomes prompted a 15-fold increase in fluorescence (164.5 FRI, 146.1 FRI, and 194.1 FRI, respectively; 10.9 FRI for EM-treated LX-2 cells) (Figure 4c). The synergistic effect induced by S80–silymarin, SMg–silymarin, and SCa–silymarin (227.3 FRI, 187.3 FRI, and 207.8 FRI, respectively) was slightly lower than that obtained with naïve LX-2 cells (18-fold increase), with DOPC–silymarin formulations (53.9 FRI), resulting also in this case in only a 5-fold increase with respect to the untreated cells (Figure 3b and Figure 4c). DLPC treatments performed only slightly better than the DOPC-based formulations (6.6 FRI) (Figure 4c).

Overall, these data suggest that PPC formulations are capable of restoring proliferative myofibroblastic LX-2 cells to a non-dividing, lipid-storing phenotype, typical of quiescent-like HSCs [2], similarly to what was described for Rol + PA treatments and their induced ADRP upregulation [43].

After LX-2 cell perpetuation, cells treated with S80–silymarin exhibited non-collagenous proteins (Figure 4b). All the other lipids stained equally for collagenous and non-collagenous proteins (Figure 4a,b and Figure S8). α-SMA expression in perpetuated LX-2 cells treated with PPC-containing liposomes decreased slightly in comparison with TGF-β_1_-treated cells (Figure 4d), thus reflecting a steady partial activated HSC state, as recently suggested in a study elucidating the fate of HSC/myofibroblasts in experimental fibrosis [58].

### 3.3. Cell Membrane Motional Order of Adherent LX-2 Cells

The rationale behind the use of two dyes lay in their different interaction with the bilayer regions. This difference is highlighted by the incubation time of the two probes used. It has been reported that the hydrophobic fluorophore DPH is located in the inner region of the phospholipid bilayer, whereas TMA-DPH is more hindered in translational and rotational motions compared to DPH [59]. Therefore, DPH estimates the motional order of the inner hydrophobic membrane region, while TMA-DPH senses changes in motional order at the interface region. TMA-DPH required 10 min to be anchored at the lipid/water interface, while the hydrophobic DPH required a longer time to be incorporated into the bilayer (2 h).

**Figure 5:**
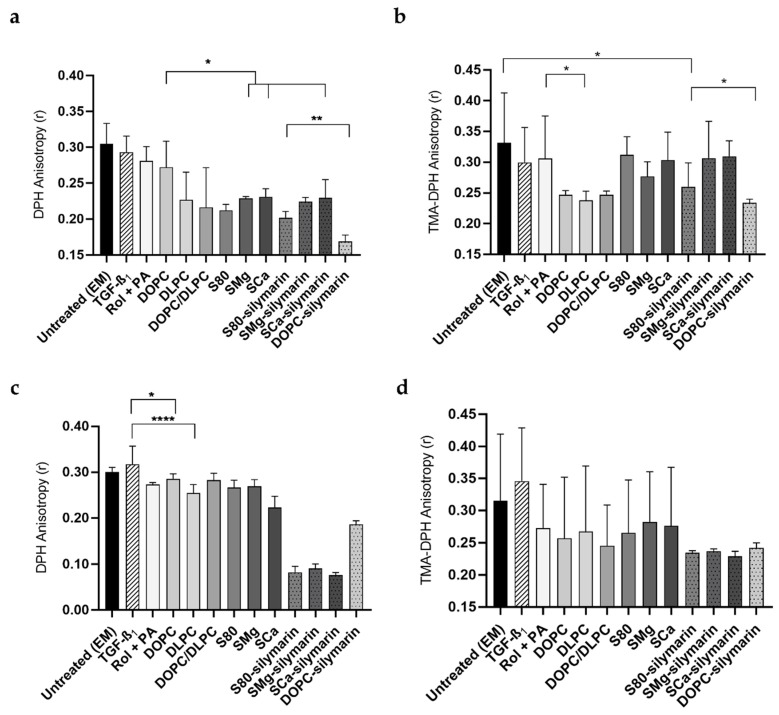
Motional order of adherent LX-2 cell membrane expressed as the anisotropy of DPH and TMA-DPH. Mean ± S.D. (*n* = 3-6). (**a**) Direct treatment of LX-2 cells. * *p* ≤ 0.05, ** *p* ≤ 0.01. Other significant differences in Table S2. (**b**) Direct treatment of LX-2 cells. * *p* ≤ 0.05. Other significant differences in Table S3. (**c**) Reversion of pLX-2 cells. * *p* ≤ 0.05, **** *p* ≤ 0.0001. Other significant differences in Table S4. (**d**) Reversion of pLX-2 cells.

In direct treatments, a decrease of DPH anisotropy correspondent to increased motional order of the inner cell membrane was observed following Rol + PA treatment when compared to the EM, in accordance with previous reports [60]. As far as phospholipid treatments were concerned, all the liposomal formulations significantly increased the motional order with respect to that observed with EM and TGF-β_1_, except for DOPC (Figure 5a). Specifically, DOPC treatments did not affect the DPH anisotropy, while a significant treatment-induced decrease (*p* ≤ 0.0001) of the measured anisotropy coefficients (r) could be observed with DLPC, S80, SMg, SCa, and silymarin liposomes (r value > 0.22) compared to EM (r value of 0.30). The ability of DLPC to be incorporated into damaged sections of the cell membrane and to increase membrane fluidity has long been proposed to be the reason behind the hepatoprotectant action of EPLs, but has never been experimentally investigated [36]. DOPC–silymarin treatment decreased r significantly from a value of 0.30 (untreated LX-2 cells, EM) to a value of 0.17.

When the amphiphilic TMA-DPH was used as a fluorescent anisotropy probe, a decrease in r value from 0.33 of untreated cells (EM) to 0.24 by treating cells directly with DLPC, DOPC, DOPC/DLPC, and DOPC–silymarin could be observed (Figure 5b). In general, the interaction of the DOPC/DLPC lipids happened more at the interfacial level, while the PPC seemed to be more active in the inner core of the plasma membrane.

S80, SMg, and SCa liposomes formulated with silymarin induced a significant decrease of the DPH r values from 0.33 (TGF-β_1_-treated) to 0.10 (Figure 5c) in pLX-2 cells. An increased motional order, although not significant, was also observed with TMA-DPH following the use of the same lipids (Figure 5d). DLPC, DOPC, DOPC/DLPC, S80, SMg, and SCa had a negligible effect on the inner membrane, while at the interface region, the effect was similar to the one produced by Rol + PA (Figure 5d).

Both membrane reporters enabled analysis of the motional order of the cell membrane as a function of the antifibrotic effect of different PPC-based formulations. Remarkably, these experiments were carried out on living adherent LX-2 cells by means of a high-throughput setup (a microplate reader equipped with polarization filters), expanding on the conventional anisotropy investigations on biological systems based on suspended cells in quartz cuvettes [60].

## 4. Conclusions

Herein, we sought to understand the antifibrotic therapeutic utility of phospholipid-based formulations in deactivating profibrogenic HSCs with an in vitro study based on the main hallmarks of quiescent and activated HSCs.

**Scheme 2:**
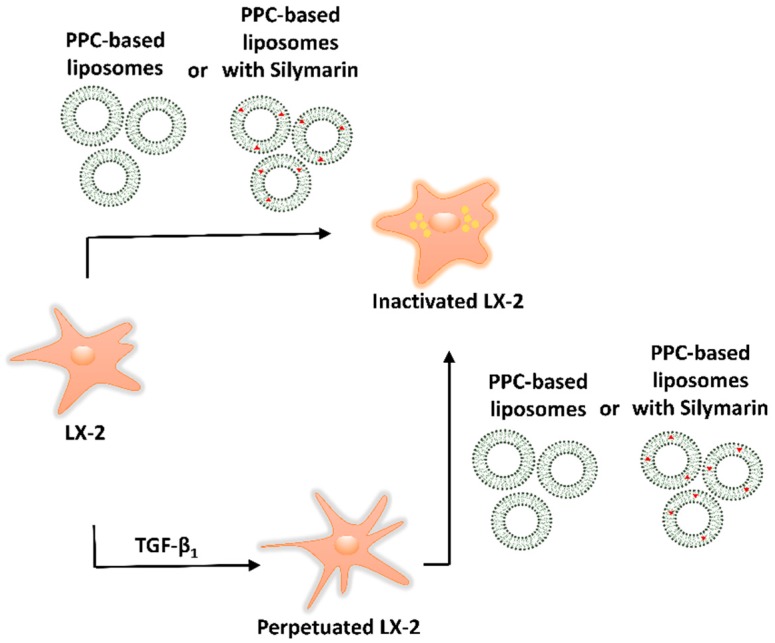
LX-2 cell phenotype modulation by phospholipid-based formulations as antifibrotic therapeutics.

The phenotype of HSCs was first modulated by optimizing known treatments with TGF-β_1_ and Rol + PA. The optimized in vitro protocols were further used for a screening of the impact of our own phospholipid-based formulations on activated LX-2 cells in absence and presence of the hepatoprotectant silymarin, as depicted in Scheme 2.

The abundance of lipid droplets, the deficiency of collagen and α-SMA, and the rise in membrane fluidity in LX-2 cells exposed to S80, SMg, or SCa suggested a reversion of HSC transdifferentiation. These modulating effects were synergistically enhanced by co-formulating the hepatoprotectant silymarin with S80. DOPC, DLPC, and DOPC/DLPC induced an increase in inner membrane fluidity, but their contributions to lipid droplet formation, collagen reduction, and α-SMA was negligible. DLPC-based formulations enhanced the breakdown of collagen, as previously reported, but did not seem to compete with the PPC-containing S80 family.

Although further biochemical studies are required, liposomes formulated with the natural phospholipid S80 and its new complexes with Mg and Ca salts, SMg and SCa, show high potential for long-term antifibrotic therapies either alone or in combination with hepatoprotectants such as silymarin. Thus, in a follow-up investigation, these three PPCs will be employed as the main components of oral dosage forms and investigated in relevant experimental models of fibrosis.

## Supplementary Materials

The following are available online at https://www.mdpi.com/1999-4923/11/12/676/s1. S80, SMg, SCa content Table S1: percentages of the different PC-species in S80, SCa and SMg; quantification of lipids; quantification of silymarin; fluorescence and phase contrast image acquisition details; Figure S1: cell viability; Figure S2: liposome characterization; Figure S3: percentage of lipid recovered after the extrusion; Figure S4: representative chromatograms of SCa phospholipid-content analysis; Figure S5: concentration of silymarin in the formulations before and after purification; Figure S6: representative chromatograms of the S80 silymarin content analysis; Figure S7: representative images of ORO and Sirius Red/Fast Green staining upon LX-2 cell direct treatment with SMg, SMg–silymarin, and SCa–silymarin; Figure S8: representative images of ORO and Sirius Red/Fast Green staining upon pLX-2 cell treatment with SMg, SMg–silymarin, and SCa–silymarin; Table S2: Tukey’s multiple comparison test of DPH anisotropy values. Direct treatment of LX-2; Table S3: Tukey’s multiple comparison test of TMA-DPH anisotropy values. Direct treatment of LX-2; Table S4: Tukey’s multiple comparison test of DPH anisotropy values. Treatment of pLX-2.
