# Supplementary material for: Synergy of Phospholipid—Drug Formulations Significantly Deactivates Profibrogenic Human Hepatic Stellate Cells
Source: Pharmaceutics. 2019 Dec 12;11(12):676. doi: 10.3390/pharmaceutics11120676 (PMC6969915; doi:10.3390/pharmaceutics11120676)
Supplement: Supplementary file 1 [file pharmaceutics-11-00676-s001.pdf]

pharmaceutics

Supplementary Materials: Synergy of Phospholipid-Drug Formulations Significantly Deactivates Pro-Fibrogenic Human Hepatic Stellate Cells

Gina Valentino, Cristina Zivko, Florian Weber, Lorine Brülisauer and Paola Luciani

Supplementary Materials and Methods

S80, SMg, SCa Content

Table S1. S80, SMg, SCa content. According to the manufacturer, the percentages of the different PC-species in S80, SCa and SMg were calculated from the area of the most intensive peaks of the LC–MS/MS chromatograms. P, palmitic acid; Le, α-linolenic acid; L, linoleic acid; O, oleic acid; S, stearic acid.

PC-species	Relative %
DLPC	33.5
PLPC	24.0
LOPC	16.0
SLPC	7.8
LLePC	7.3
POPC	4.6
DOPC	2.3
PLePC	2.1
SOPC	1.1
DLePC	0.4
DPPC	0.4
PeLPC	0.3

Quantification of Lipids

The lipid content of the phospholipid-based formulations was determined by HPLC (1260 Infinity II, Agilent Technologies, Santa Clara, CA, USA), equipped with a pump, an autosampler, a charged aerosol detector (CAD) (Corona™, ESA Bioscience, Chelmsford, MA, USA), and a nitrogen generator (outlet pressure 36 psi, ESA Bioscience). The evaluation-software used was ChemStation OpenLAB CDS, version 2.15 (Agilent Technologies). An InfinityLab Poroshell 120EC-C18 column (C18, 3 × 100 mm, 2.7 µm; Agilent Technologies) was used at 30 °C, while the samples were kept at 8 °C. The injected volume was 5 µL and the flow rate was set at 0.5 mL/min. A combination of two different solvents was used to create the mobile phase: Solvent A was (ACN + 0.05% *v*/*v* TFA)/(H_2O + 0.05% *v*/*v* TFA) (90:10, *v*/*v*), and solvent B was MeOH + 0.05% *v*/*v* TFA. The elution of the samples started isocratic with solution A/solution B (60:40, *v*/*v*) for 25 min followed by a linear gradient of solution B over 15 min (40–100% *v*/*v*). Prior to HPLC analysis, the lipids were diluted 1:49 (*v*/*v*) with methanol to match the concentration range of the calibration curves (0.01–0.75 mg/mL) and to destroy the vesicular structure of the liposomes. The sample was then further diluted 1:1 (*v*/*v*) with palmitic acid in methanol (conc. 0.4 mg/mL, internal standard). The liposomal formulations were tested before and after the extrusion. For the standard curve, standard solutions of each lipid were prepared in MeOH. The concentration range was chosen for each lipid, based on the initial concentration used for the preparation of liposomes and ranged from 0.01 to 0.75 mg/mL. The mean peak area ± standard deviation (S.D.) was calculated and plotted against the known concentration of the standard.

Quantification of Silymarin

For the quantification of silymarin, a modified version of the method described in the European Pharmacopoeia 8.8 was performed. Briefly, a HPLC system (Dionex, Thermo Fisher Scientific, Waltham, MA, USA) equipped with a quaternary pump P680, column oven, autosampler ASI 100, and a DAD-UV detector UVD430U was used. A MN Nucleosil column (C18, 3.0 × 125 mm, 5 µm, Macherey Nagel, Düren, Germany) was used as the stationary phase at 30 °C. For sample preparation, 10 µL of liposome dispersion (25 mM total lipid content) and 90 µL MeOH were mixed and homogenized in an ultrasound bath for 10 min at room temperature. The injection volume was 20 µL and the flow rate was 1 mL/min. The detection wavelength was set to 285 nm. A combination of two different solvents was used to create the mobile phase: Solvent A was MeOH/H_2O (35:65, *v/v*) + 0.05% *v/v* H_3PO_4, and solvent B was MeOH/H_2O (50:50, *v/v*) + 0.05% *v/v* H_3PO_4. Silymarin was eluted with a linear gradient of solution B over 19.5 min (0-69.6% *v/v*) followed by a 1 min isocratic elution with solution A/solution B (30.4:69.6, *v/v*). After 20.5 min solution A was increased to 100% until 22 min. Evaluation was performed with Chromeleon 6.0. Encapsulation efficiency (EE %) was calculated using below formula:

$$\text{Encapsulation efficiency (\%)} = (Dt/Di) \times 100, \quad (1)$$

where Dt is the total amount of drug in the liposomes and Di is the total quantity of drug in added initially in the liposomes.

Fluorescence and Phase Contrast Image Acquisition Details

Fluorescence and phase contrast images acquisition was performed using a Nikon Ti-U (Nikon Instruments, Melville, NY, USA) inverted microscope coupled to Nikon cameras DS-Qi2 and DFK 33UX174.

Objectives used for ORO staining: Plan Fluor EL WD 20x Ph 1 ADL with numerical aperture of 0.45 and refractive index 1.0.; Plan Fluor EL WD 40x Ph 1 ADL with numerical aperture 0.6 and refractive index 1.0. DAPI filter (ex 360, em 460), TexRed filter (ex 560, em 645) were used. The fluorescent binary area and the object count were automatically detected with the NIS Elements software v. 5.00 and exported.

Sirius Red/Fast Green staining: objectives Plan Fluor EL WD 40x Ph 1 ADL with numerical aperture 0.6 and refractive index 1.0.

α-SMA expression analysis: objective Plan Fluor EL WD 20x Ph 1 ADL with numerical aperture of 0.45 and refractive index 1.0. DAPI filter (ex 360, em 460), Cy-5 filter (ex 640, em 670) were used. The fluorescent binary area and the object count were automatically detected with the NIS Elements software v. 5.00 and exported.

Supplementary Results

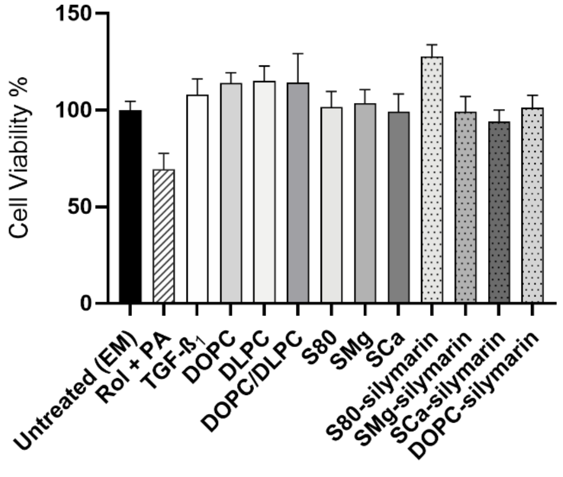

Figure S1. Cell viability (%) using CCK8 assay examined at 24 h. Mean ± S.D. (*n* = 3).

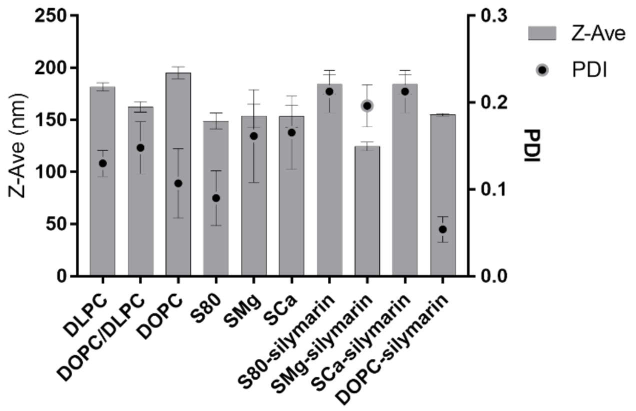

Figure S2. Size and polydispersity index (PDI) of liposomes determined by dynamic light scattering. Mean ± S.D. (n = 3–9).

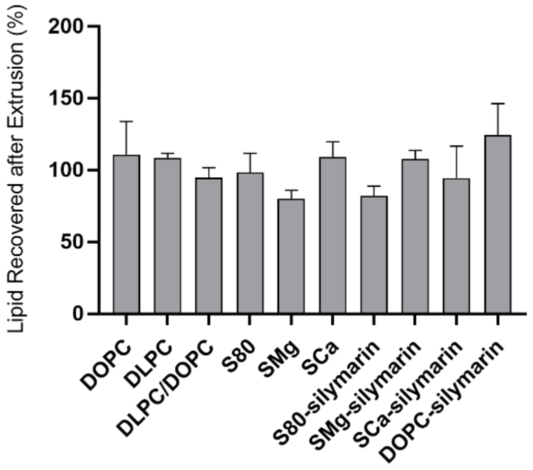

Figure S3. Percentage of lipid recovered after the extrusion determined by HPLC. Mean ± S.D. (n = 3).

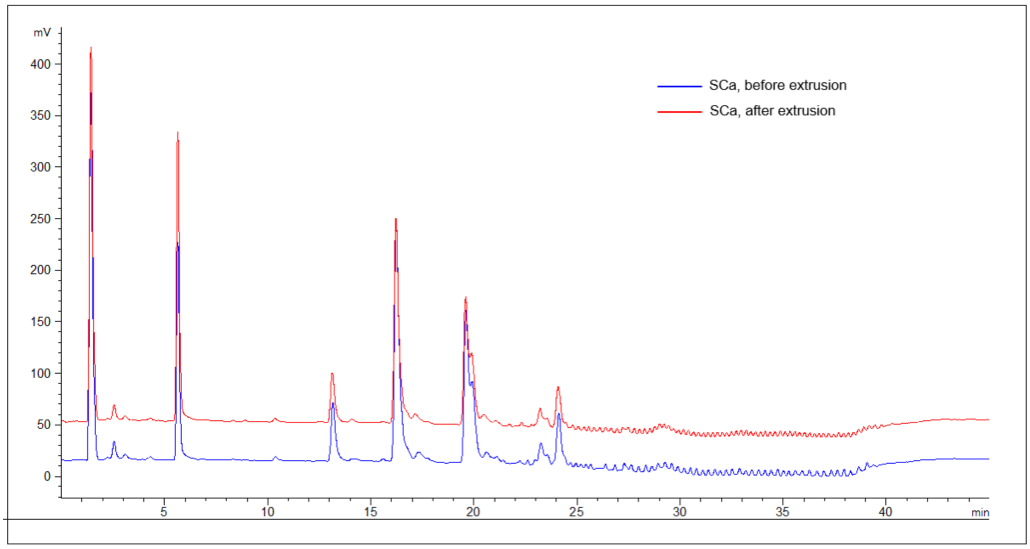

Figure S4. Displayed is an overlay of two representative chromatograms of SCa phospholipid-content before and after extrusion obtained by HPLC-CAD (10% offset of the detector signal, 0% offset in retention time).

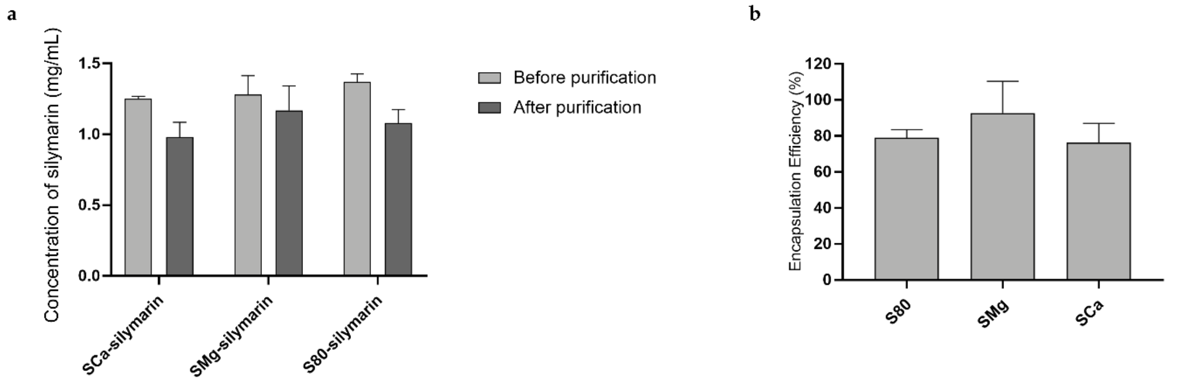

Figure S5. (a) Concentration of silymarin in the formulations before and after the purification determined by HPLC. **(b)** Percentage of silymarin encapsulation efficiency determined by HPLC. Mean ± S.D. (n = 3).

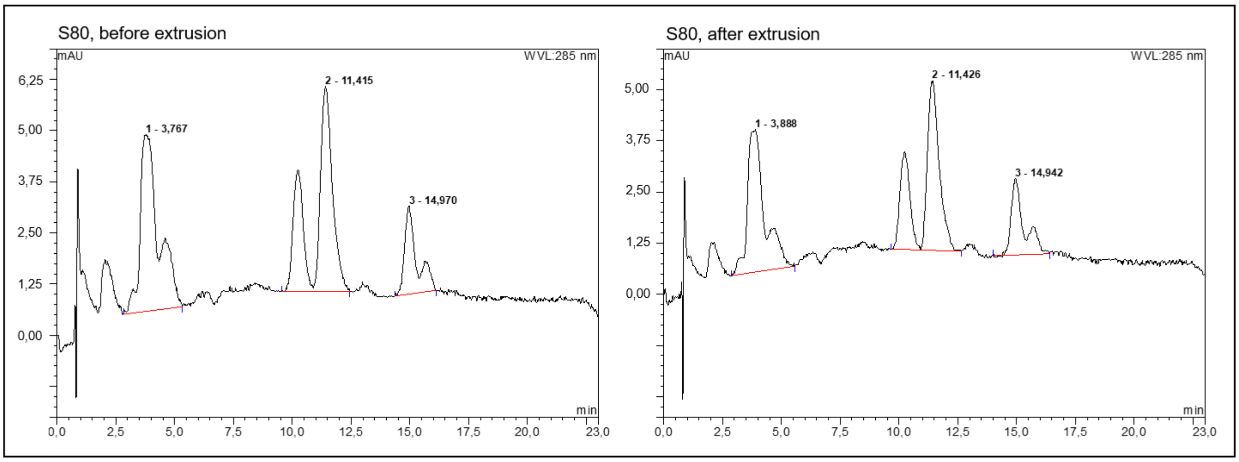

Figure S6. Displayed are two representative chromatograms of the silymarin content in S80–silymarin liposomes before and after extrusion. Analysis obtained by HPLC-UV.

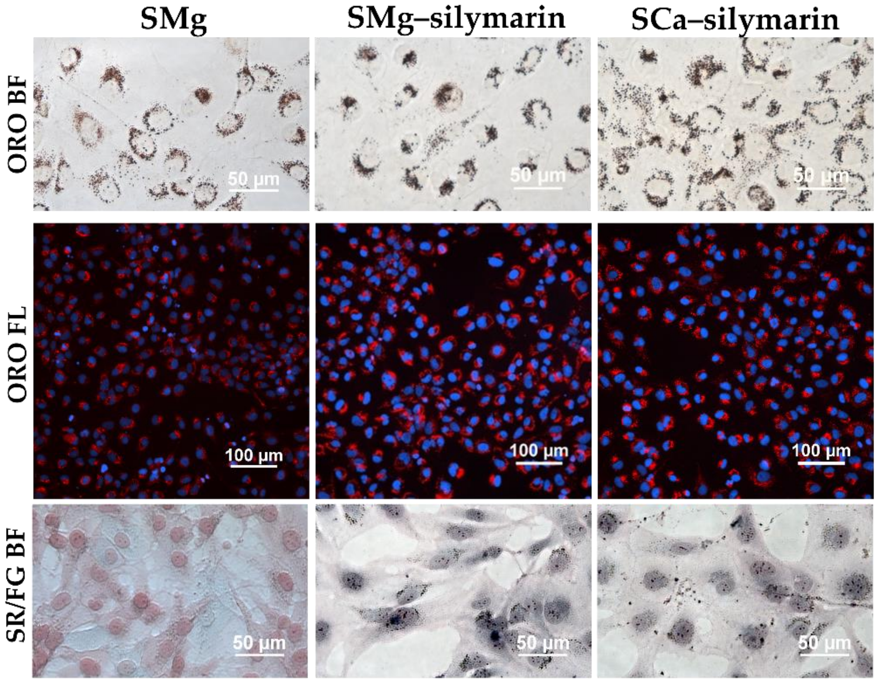

Figure S7. LX-2 cells directly treated with SMg, SMg–silymarin and SCa–silymarin. Representative microscopy images of lipid droplets (ORO staining) and collagen (Sirius Red/Fast Green staining). Lipid droplets in bright

field (ORO BF) appear as brown spots and in fluorescence (ORO FL) as red spots (nuclei stained with blue DAPI). The presence of collagen is observed in purple in bright field SR/FG BF); while non-collagenous proteins are stained green.

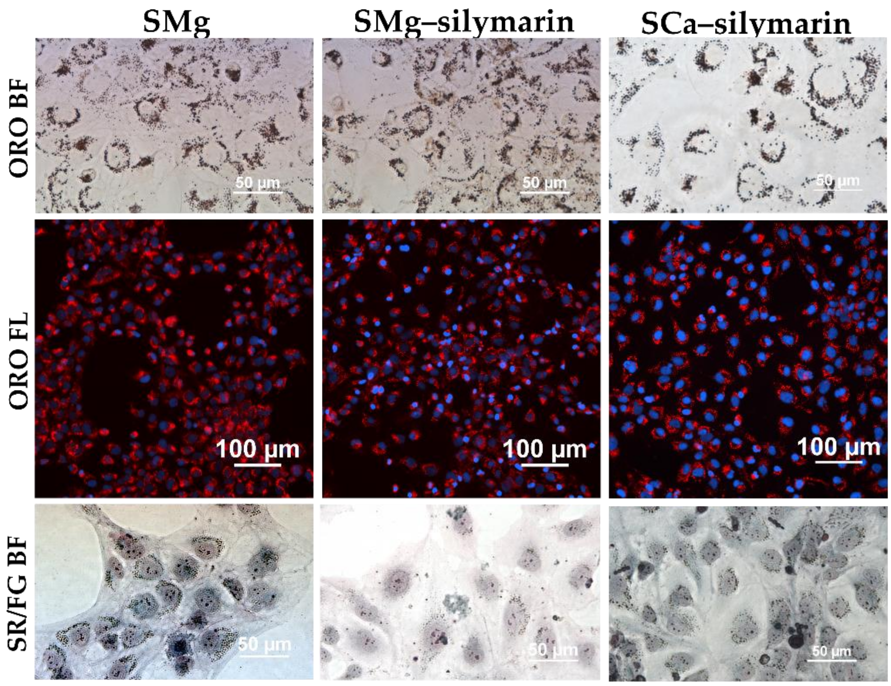

Figure S8. pLX-2 cells treated with SMg, SMg–silymarin and SCa–silymarin. Representative microscopy images of lipid droplets (ORO staining) and collagen (Sirius Red/Fast Green staining). Lipid droplets in bright field (ORO BF) appear as brown spots and in fluorescence (ORO FL) as red spots (nuclei stained with blue DAPI). The presence of collagen is observed in purple in bright field SR/FG BF); while non-collagenous proteins are stained green.

Table S2. Tukey's multiple comparison test of DPH anisotropy values. Direct treatment of LX-2.

Multiple comparison	Summary	P Value
Untreated (EM) vs. DLPC	****	<0.0001
Untreated (EM) vs. DOPC/DLPC	****	<0.0001
Untreated (EM) vs. S80	****	<0.0001
Untreated (EM) vs. SMg	****	<0.0001
Untreated (EM) vs. SCa	****	<0.0001
Untreated (EM) vs. S80–silymarin	****	<0.0001
Untreated (EM) vs. SMg–silymarin	****	<0.0001
Untreated (EM) vs. SCa–silymarin	****	<0.0001
Untreated (EM) vs. DOPC–silymarin	****	<0.0001
TGF-ß1 vs. DLPC	****	<0.0001
TGF-ß1 vs. DOPC/DLPC	****	<0.0001
TGF-ß1 vs. S80	****	<0.0001
TGF-ß1 vs. SMg	****	<0.0001
TGF-ß1 vs. SCa	****	<0.0001
TGF-ß1 vs. S80–silymarin	****	<0.0001
TGF-ß1 vs. SMg–silymarin	****	<0.0001
TGF-ß1 vs. SCa–silymarin	****	<0.0001
TGF-ß1 vs. DOPC–silymarin	****	<0.0001
Rol + PA vs. DLPC	****	<0.0001
Rol + PA vs. DOPC/DLPC	****	<0.0001

Rol + PA vs. S80	****	<0.0001
Rol + PA vs. SMg	****	<0.0001
Rol + PA vs. SCa	***	<0.0001
Rol + PA vs. S80–silymarin	****	<0.0001
Rol + PA vs. SMg–silymarin	****	<0.0001
Rol + PA vs. SCa–silymarin	****	<0.0001
Rol + PA vs. DOPC–silymarin	****	<0.0001
DLPC vs. DOPC	**	0.0096
DLPC vs. DOPC-Silymarin	****	<0.0001
DOPC vs. DOPC/DLPC	***	0.0003
DOPC vs. S80	****	<0.0001
DOPC vs. SMg	*	0.0176
DOPC vs. SCa	*	0.0323
DOPC vs. S80–silymarin	****	<0.0001
DOPC vs. SMg–silymarin	**	0.0046
DOPC vs. SCa–silymarin	*	0.0240
DOPC vs. DOPC–silymarin	****	<0.0001
DOPC/DLPC vs. DOPC–silymarin	***	0.0004
S80 vs. DOPC–silymarin	**	0.0023
SMg vs. DOPC–silymarin	****	<0.0001
SCa vs. DOPC–silymarin	****	<0.0001
SMg–silymarin vs. DOPC–silymarin	****	<0.0001
SCa–silymarin vs DOPC–silymarin	****	<0.0001

Table S3. Tukey's multiple comparison test of TMA-DPH anisotropy values. Direct treatment of LX-2.

**Multiple comparisons**	**Summary**	**P Value**
Untreated (EM) vs. DLPC	***	0.0004
Untreated (EM) vs. DOPC	**	0.0024
Untreated (EM) vs. DOPC/DLPC	**	0.0023
Untreated (EM) vs. S80–silymarin	*	0.0239
Untreated (EM) vs. DOPC–silymarin	****	<0.0001
TGF-ß$_1$ vs. DOPC–silymarin	**	0.0028
Rol + PA vs. DLPC	*	0.0384
Rol + PA vs. DOPC–silymarin	**	0.0014
DLPC vs. S80	*	0.0477
S80 vs. DOPC–silymarin	**	0.0044
SCa vs. DOPC–silymarin	*	0.0139
SMg–silymarin vs. DOPC–silymarin	*	0.0125
SCa–silymarin vs. DOPC–silymarin	**	0.0073

Table S4. Tukey's multiple comparison test of DPH anisotropy values. Treatment of pLX-2.

Multiple comparisons	Summary	P Value
Untreated (EM) vs. DLPC	****	<0.0001
Untreated (EM) vs. S80	**	0.0058
Untreated (EM) vs. SMg	*	0.0161
Untreated (EM) vs. SCa	****	<0.0001
Untreated (EM) vs. S80–silymarin	****	<0.0001
Untreated (EM) vs. SMg–silymarin	****	<0.0001
Untreated (EM) vs. SCa–silymarin	****	<0.0001

Untreated (EM) vs. DOPC–silymarin	****	<0.0001
TGF-ß$_1$ vs. Rol + PA	***	0.0006
TGF-ß$_1$ vs. DLPC	****	<0.0001
TGF-ß$_1$ vs. DOPC	*	0.0161
TGF-ß$_1$ vs. DOPC/DLPC	**	0.0058
TGF-ß$_1$ vs. S80	****	<0.0001
TGF-ß$_1$ vs. SMg	****	<0.0001
TGF-ß$_1$ vs. SCa	****	<0.0001
TGF-ß$_1$ vs. S80–silymarin	****	<0.0001
TGF-ß$_1$ vs. SMg–silymarin	****	<0.0001
TGF-ß$_1$ vs. SCa–silymarin	****	<0.0001
TGF-ß$_1$ vs. DOPC–silymarin	****	<0.0001
Rol + PA vs. SCa	****	<0.0001
Rol + PA vs. S80–silymarin	****	<0.0001
Rol + PA vs. SMg–silymarin	****	<0.0001
Rol + PA vs. SCa–silymarin	****	<0.0001
Rol + PA vs. DOPC–silymarin	****	<0.0001
DLPC vs. DOPC	**	0.0033
DLPC vs. DOPC/DLPC	*	0.0112
DLPC vs. SCa	**	0.0026
DLPC vs. S80–silymarin	****	<0.0001
DLPC vs. SMg–silymarin	****	<0.0001
DLPC vs. SCa–silymarin	****	<0.0001
DLPC vs. DOPC–silymarin	****	<0.0001
DOPC vs. SCa	****	<0.0001
DOPC vs. S80–silymarin	****	<0.0001
DOPC vs. SMg–silymarin	****	<0.0001
DOPC vs. SCa–silymarin	****	<0.0001
DOPC vs. DOPC–silymarin	****	<0.0001
DOPC/DLPC vs. SCa	****	<0.0001
DOPC/DLPC vs. S80–silymarin	****	<0.0001
DOPC/DLPC vs. SMg–silymarin	****	<0.0001
DOPC/DLPC vs. SCa–silymarin	****	<0.0001
DOPC/DLPC vs. DOPC–silymarin	****	<0.0001
S80 vs. SCa	****	<0.0001
S80 vs. S80–silymarin	****	<0.0001
S80 vs. SMg–silymarin	****	<0.0001
S80 vs. SCa–silymarin	****	<0.0001
S80 vs. DOPC–silymarin	****	<0.0001
SMg vs. SCa	****	<0.0001
SMg vs. S80–silymarin	****	<0.0001
SMg vs. SMg–silymarin	****	<0.0001
SMg vs. SCa–silymarin	****	<0.0001
SMg vs. DOPC–silymarin	****	<0.0001
SCa vs. S80–silymarin	****	<0.0001
SCa vs. SMg–silymarin	****	<0.0001
SCa vs. SCa–silymarin	****	<0.0001
SCa vs. DOPC–silymarin	***	0.0003
S80–silymarin vs. DOPC–silymarin	****	<0.0001
SMg–silymarin vs. SCa- Silymarin	ns	0.7989

SMg–silymarin vs. DOPC–silymarin	****	<0.0001
SCa–silymarin vs. DOPC–silymarin	****	<0.0001
